# A machine learning approach using endpoint adjudication committee labels for the identification of sepsis predictors at the emergency department

**DOI:** 10.1186/s12873-022-00764-9

**Published:** 2022-12-23

**Authors:** Michael S. A. Niemantsverdriet, Titus A. P. de Hond, Imo E. Hoefer, Wouter W. van Solinge, Domenico Bellomo, Jan Jelrik Oosterheert, Karin A. H. Kaasjager, Saskia Haitjema

**Affiliations:** 1 Central Diagnostic Laboratory, University Medical Center Utrecht, Utrecht University, Room Number G03.551, Heidelberglaan 100, 3584 CX Utrecht, The Netherlands; 2 SkylineDx, Rotterdam, The Netherlands; 3 Department of Internal Medicine and Acute Medicine, University Medical Center Utrecht, Utrecht University, Utrecht, the Netherlands; 4 Department of Internal Medicine, Infectious Diseases, University Medical Center Utrecht, Utrecht University, Utrecht, the Netherlands

**Keywords:** Sepsis, Electronic health records, Emergency department, Machine learning, Endpoint adjudication

## Abstract

Accurate sepsis diagnosis is paramount for treatment decisions, especially at the emergency department (ED). To improve diagnosis, clinical decision support (CDS) tools are being developed with machine learning (ML) algorithms, using a wide range of variable groups. ML models can find patterns in Electronic Health Record (EHR) data that are unseen by the human eye. A prerequisite for a good model is the use of high-quality labels. Sepsis gold-standard labels are hard to define due to a lack of reliable diagnostic tools for sepsis at the ED. Therefore, standard clinical tools, such as clinical prediction scores (e.g. modified early warning score and quick sequential organ failure assessment), and claims-based methods (e.g. ICD-10) are used to generate suboptimal labels. As a consequence, models trained with these “silver” labels result in ill-trained models. In this study, we trained ML models for sepsis diagnosis at the ED with labels of 375 ED visits assigned by an endpoint adjudication committee (EAC) that consisted of 18 independent experts. Our objective was to evaluate which routinely measured variables show diagnostic value for sepsis. We performed univariate testing and trained multiple ML models with 95 routinely measured variables of three variable groups; demographic and vital, laboratory and advanced haematological variables. Apart from known diagnostic variables, we identified added diagnostic value for less conventional variables such as eosinophil count and platelet distribution width. In this explorative study, we show that the use of an EAC together with ML can identify new targets for future sepsis diagnosis research.

**Supplementary Information:**

The online version contains supplementary material available at 10.1186/s12873-022-00764-9.

## Introduction

Sepsis is a severe clinical illness, defined as a life-threatening organ dysfunction caused by a dysregulated host response to infection [1]. The diagnosis of sepsis still remains challenging due to the heterogeneity of clinical symptoms, and the lack of robust diagnostic tests [2–4]. Early recognition of sepsis in the emergency department (ED) can be particularly difficult as only limited time and measurements are available for accurate diagnosis. Therefore, numerous tools have been proposed in literature, such as the quick sequential organ failure assessment (qSOFA) score, early warnings scores (e.g. modified early warning score (MEWS)), and biomarkers (e.g. CD64, red blood cell distribution width and pancreatic stone protein) [1, 5–8]. However, as most of these tools were developed for prognostic purposes, using them for diagnostic endpoints often results in disappointing results [9].

Machine learning (ML) approaches may help doctors to integrate routine care data from EHR databases in diagnostic models. As some ICU databases are publicly available, many studies already used ML to develop diagnostic models for sepsis in the ICU [10, 11]. Though some of these models show high discriminative power, none of them have been broadly implemented in the clinic yet, because of important limitations, including lack of validation and differences in the used definition for sepsis, thereby limiting generalizability [12, 13].

The lack of reliable labels is a major challenge in sepsis research at the ED. By lack of a gold standard, diagnostic tools (e.g. MEWS, qSOFA) and claims-based methods (e.g. ICD-10) are currently used to generate labels for sepsis modelling. Nonetheless, these labelling methods are known to be suboptimal [14]. As the quality of the input defines the eventual quality of the model, i.e. the diagnostic performance, using high quality outcome labels is an essential factor for developing accurate models with ML algorithms. A good alternative is an endpoint adjudication committee (EAC) which is a proven method to gain consensus on outcome, and define labels for machine learning practices [12, 14].

For this study, we used ML and EAC generated labels to explore 95 variables from multiple types of variable groups; demographic and vital functions (1), laboratory tests (2) and advanced haematological variables from the *Abbott CELL-DYN* sapphire analyser (3), to find additional value in routinely stored EHR data in the ED. We estimated the importance of each variable to identify what variables are most discriminative for sepsis, generating hypotheses for future research.

## Methods

### Study design and setting

We included patients from the SPACE-cohort (*SePsis in the ACutely ill patients in the Emergency department*) in the University Medical Centre Utrecht (UMCU) [7]. In brief, ED patients with a suspicion of infection presenting who presented at either internal medicine or its subspecialties (endocrinology, geriatrics, haematology, immunology, infectious disease, nephrology, oncology, rheumatology and vascular medicine) were included in SPACE, no specific exclusion criteria were used. Suspicion of infection was automatically documented by the treating physician, via the electronic health system. An independent physician validated whether the suspicion of infection of each patient by evaluating anti-microbial treatment, microbiological culturing or other signs of infection. Demographic data and physiological variable measurements collected during ED visit were documented and validated by a team of physicians. In this study we used a subset of the SPACE cohort, namely all 375 ED visits that had been labelled for sepsis by the EAC with a blood sample within the first hours of ED visit.

### Endpoint adjudication committee

An EAC with 18 independent experts was formed. The EAC consisted of ED specialists, internists (subspecialty infectiology or acute medicine) and ICU specialists. All EHR data including the ED, follow-up in the hospital and after discharge, was made available to the EAC. Members of the EAC were instructed to identify sepsis based on international guidelines (Sepsis-3). All available medical data was assessed by two independent experts. In case of disagreement, an additional expert was consulted followed by a majority vote to generate the final label.

### Data collection and pre-processing

ED patients suspected of infection are routinely evaluated by a specific panel of laboratory variables related to infection measured in blood ("internal medicine laboratory panel” or “sepsis panel”). Standard haematology variables included in the panel are routinely collected by the *Abbott CELL-DYN* Sapphire haematology analyser (Abbott Diagnostics, Santa Clara, CA, USA) [13, 15]. In addition, the *Abbott CELL-DYN* measures advanced haematology variables that are automatically measured and stored in the laboratory information system, yet not shown in the electronic health record and available for research purposes. The Sapphire is a 5-diff haematological automated cell counter system equipped with an integrated 488-nm blue diode laser and uses multiple techniques, such as electrical impedance, spectrophotometry and laser light scattering, to measure morphological characteristics of leukocytes, RBCs, and platelets for classification and enumeration [16].

Laboratory and advanced haematology data were extracted for SPACE patients from the Utrecht Patient Oriented Database (UPOD) [17]. In brief, UPOD is an infrastructure of relational databases comprising data on patient characteristics, hospital discharge diagnoses, medical procedures, medication orders and laboratory tests for all patients treated at the UMCU since 2004. Only ED visits with measurements for both the laboratory panel and the advanced haematology variables were selected. We removed variables that showed high correlation (Pearson correlation > 0.8) to reduce multicollinearity. We did this for two reasons: (1) removal of highly correlated variables would remove noise from our results (2), advanced ML algorithms can arbitrarily select one of two highly correlated variables, thereby affecting the analysis what variables are important in the models. Moreover, variables with a low number of unique values (N < 5) were removed. Missing measurements were imputed with the variable’s median.

### Model development

We were interested in what variables, and in particular what variable groups, had high discriminative accuracy. We used two approaches to identify predictors from the following three variable groups: demographic and vital, laboratory, and advanced haematology variables (Supplemental Table 1). First, we performed univariate analysis by fitting a logistic regression for each individual variable. To evaluate the discriminative power of each variable’s model, we computed the receiver operator characteristic curve (ROC) and precision recall curve (PRC), and subsequently the area under the ROC curve (AUROC). The ROC shows the trade-off between sensitivity and specificity, whereas the PRC shows the trade-off between the sensitivity and precision. In our study, the latter is more of interest, as we are more interested in identifying sepsis cases (sensitivity) and how certain we are of our prediction (precision). Secondly, we trained multiple ML models with the three variable groups. As there is no machine learning algorithm that ‘rules over all’ (no free lunch theorem) [18], we evaluated the diagnostic performance by training three machine learning models; logistic regression (LR), L1 regularization (L1) and Random Forest (RF) [19–21]. Both LR and L1 are able to find linear associations between the predictors and outcome, though L1 is able to reduce the coefficients of unimportant predictors to zero, thereby reducing the number of predictors. The RF algorithm trains a number of decision trees that, in comparison to LR and L1, are able to identify non-linear associations between predictors and outcome.

**Table 1:** Characteristics of all 375 emergency department (ED) visits labelled by the endpoint adjudication committee

	No sepsis (*N* = 300)	Sepsis (*N* = 75)
Unique patients, count	271	71
Age [IQR]	60.0 [45.0, 69.0]	65.0 [56.0, 72.0]
Sex, male count (%)	159 (53.0%)	38 (50.7%)
Days symptoms prior ED visit, days [IQR]	3.0 [2.0, 5.0]	2.0 [1.0, 3.0]
Charlson Comorbidity Index	4.0 [2.0, 6.0]	5.0 [4.0, 7.0]
qSOFA		
0	235 (78.3%)	20 (26.7%)
1	60 (20.0%)	32 (42.7%)
2	5 (1.7%)	18 (24.0%)
3	0 (0.0%)	5 (6.7%)
MEWS (IQR)	1.0 [0.0, 3.0]	5.0 [3.0, 6.5]
Immuno-compromised, count (%)	114 (38.1%)	21 (28.0%)
ED specialty,		
Haematology	52 (17.3%)	8 (10.7%)
Internal Medicine	93 (31.0%)	33 (44.0%)
Nephrology	58 (19.3%)	10 (13.3%)
Oncology	54 (18.0%)	15 (20.0%)
Other	43 (14.3%)	9 (12.0%)
Length of stay, days (IQR)	0.0 [0.0, 4.0]	7.0 [3.5, 12.5]
Primary infection noted in discharge letter, count (%)		
Cardiovascular	3 (0.7%)	4 (5.3%)
Intra-abdominal	40 (13.3%)	6 (8.0%)
No infection	26 (8.7%)	3 (4.0%)
Other	21 (7.0%)	3 (4.0%)
Respiratory tract infection	154 (51.3%)	37 (49.3%)
Skin Infection	16 (5.3%)	4 (5.3%)
Unknown	3 (1.0%)	0 (0.0%)
Urinary tract infection	37 (12.3%)	18 (24.0%)
Broad spectrum antibiotics, count (%)	52 (17.3%)	51 (68.0%)
ICU admission*, count (%)	8 (2.7%)	10 (15.2%)
In-hospital mortality admission, count (%)	4 (1.3%)	11 (14.7%)

qSOFA was computed using the sepsis-3 criteria [22]. Both immunocompromised and broad-spectrum antibiotics definitions are in line with UMCU protocols and explained in Supplemental Table 3

*ICU* Intensive care unit

^*^ Only patients that were applicable for ICU admission are shown

### Double loop cross validation to estimate model performance

Training classifiers with too many variables on a small cohort may result in the identification of cohort-specific patterns that may reduce the generalizability of the results, also known as overfitting [23, 24]. Model performance was therefore assessed with k-fold cross validation (CV). With CV, all data is split into *k* partitions (folds) of which in sequence *k-1* folds are used for training and 1 for testing the performance, resulting in *k* out-of-sample test estimates. To increase the model’s performance, we optimized several hyperparameters, such as the number of trees of the RF algorithm. Optimizing hyperparameters and creating a model with the same training data does not allow for a valid independent estimate of the model’s accuracy. Therefore, we applied a double loop cross validation (DLCV) scheme, also known as nested CV, to optimize several hyperparameters for each model (Supplemental Fig. 1, Supplemental Table 2). In brief, the *k-1* folds used for training the algorithm are first used in the inner CV scheme to optimize the hyperparameters, after which the best hyperparameter configuration is used in the outer CV to train the model on the same *k-1* training folds. Finally, the model is tested on the *k* fold (hold-out test set) to obtain an independent estimate of the model’s accuracy. The DLCV scheme was run once.

**Fig. 1:**
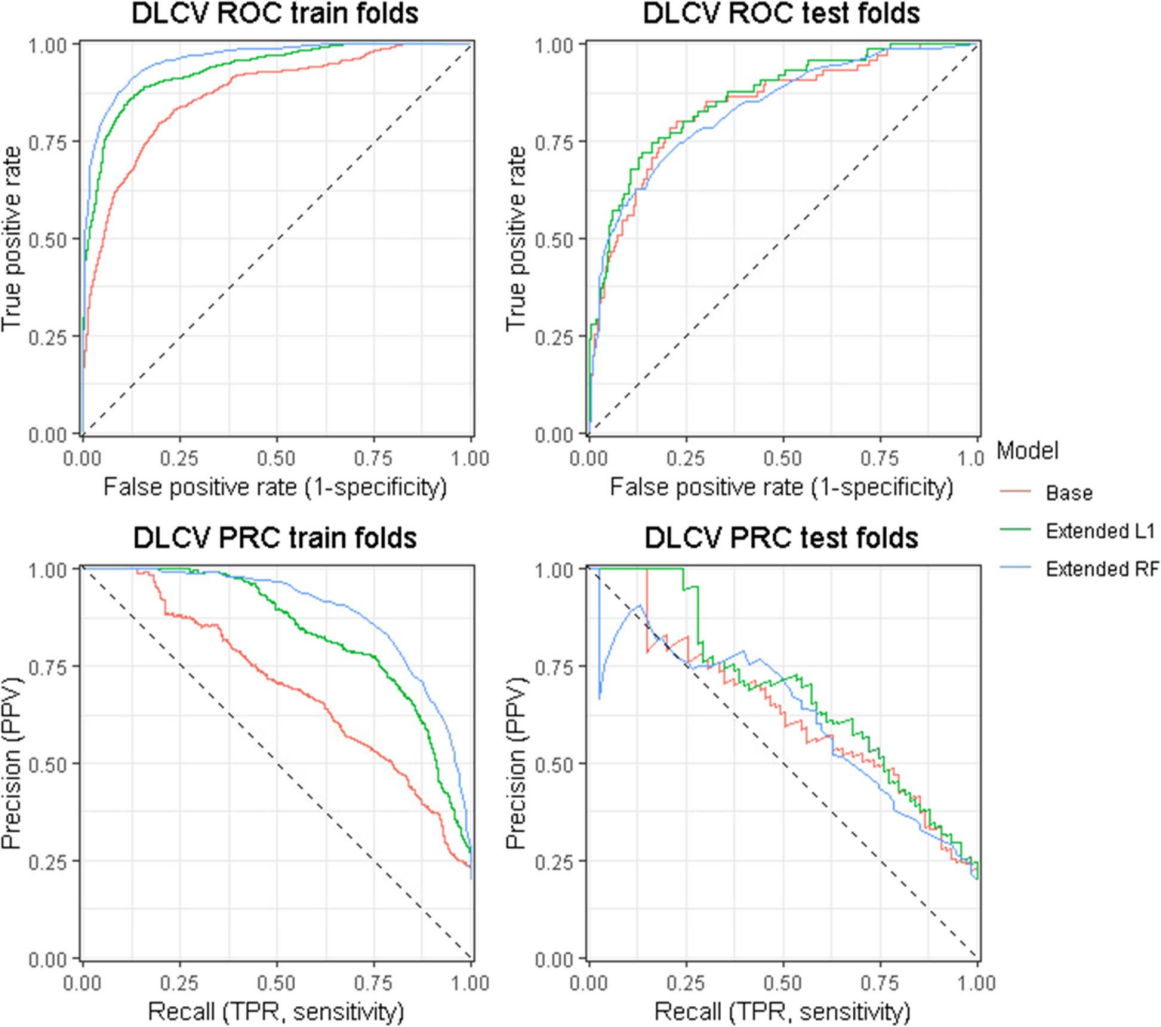
Receiver operator characteristic (ROC) and precision recall (PRC) curves of the three models with the best performance of each model configuration in the double loop cross validation (DLCV) scheme. Three model configurations are shown; base (logistic regression on demographic and vital data), extended L1 (lasso on demographic, vital, laboratory and sapphire data) and extended RF (random forest on demographic, vital, laboratory and sapphire data). For each of the three model configurations, ten models were trained on different data splits in the tenfold DLCV scheme. During each iteration, predictions were computed with on both training and test folds. Both ROC and PRC curves were drawn with the aggregated train and test data over 10 folds for each model configuration

**Table 2:** Average train and test AUC performance of models trained in the double loop cross validation pipeline

	Demographic and vital (*P* = 9)		Laboratory (*P* = 10)		Advanced haematology (*P* = 51)		All (*P* = 70)	
	Train	Test	Train	Test	Train	Test	Train	Test
Logistic regression	0.87 (0.86–0.89)	0.84 (0.79–0.89)	0.78 (0.76–0.80)	0.71 (0.64–0.77)	0.90 (0.89–0.91)	0.66 (0.59–0.73)	0.99 (0.99–0.99)	0.73 (0.66–0.81)
Lasso	0.87 (0.85–0.88)	0.84 (0.79–0.89)	0.77 (0.75–0.79)	0.71 (0.64–0.80)	0.82 (0.82–0.85)	0.77 (0.71–0.83)	0.94 (0.93–0.95)	0.85 (0.80–0.95)
Random Forest	0.90 (0.88–0.91)	0.83 (0.76–0.90)	0.88 (0.86–0.89)	0.69 (0.62–0.77)	0.91 (0.90–0.92)	0.76 (0.69–0.83)	0.96 (0.95–0.97)	0.84 (0.78–0.89)

P denotes the number of variables in each model set. 95% confidence intervals are shown in parentheses for each model and data configuration

### Model performance and variable importance

Discriminative performance between classifiers was assessed by comparing both the ROC and PRC curves. Also, performance of the 10 models trained in the DLCV for each algorithm was estimated by computing the AUROC and computing the 95% confidence interval (95% CI) with the package *cvAUC* [25]. Variable importance of LR was assessed with the estimated coefficient. For the L1, the number of times a variable was selected in the ten iterations of the DLCV and the associated coefficients were reported. Shapley values were calculated for the RF to investigate variable importance among the several models and datasets. The Shapley value method takes the sepsis labelled patients along with the non-sepsis patients, and assesses to what extent differences in variables between the two groups contribute to the model’s output. Mean and standard deviation are shown for normally distributed variables whereas median and inter-quartile range (IQR) are shown for non-normally distributed variables. All analyses were done in *RStudio* (2021.09.0) using R version 4.1.2 [26].

## Results

### Sepsis patients were older and more often male

Between 2018–01-17 and 2018–04-07 we included 375 ED visits from 335 patients with available demographic, vital and laboratory data (Table 1, Supplemental Table 1). Of all visits, 75 (20.0%) visits were labelled as having sepsis by the EAC. There were slightly more males in both sepsis (53.0%) and non-sepsis (50.7%) groups. Patients with sepsis were slightly older (median of 65.0 versus 60.0 years old) and had worse outcome; longer median length of stay (7.0 versus 0.0 days), higher ICU admission (15.2% versus 2.7%) and mortality (14.7% versus 1.4%). 4% of sepsis patients did not have an infection according to the treating physician, but the EAC did label these patients as having sepsis.

### Advanced haematology variables are predictive for sepsis

Supplemental Table 2 shows the discriminative power (AUROC) of all 95 variables used in this study. Of the vital variables, both respiratory rate (rr) and heart rate (hr) showed the highest discriminative performance, AUROC of 0.75. CRP showed the highest performance of the lab variables with an AUROC of 0.75 as well. Five advanced haematology variables; percentage of banded granulocytes (pbnd), banded granulocytes count (bnd), percentage neutrophilic granulocytes (pneu), percentage of immature granulocytes (pig) and immature granulocytes (ig), all had similar performances of AUROC values between 0.68 – 0.70.

### Combining variable groups improves sepsis diagnostic performance

After removing the collinear variables (*N* = 25) and two variables with low number of unique values (nrbc, pnrbc), 70 variables from the three variable groups were used to train models in the DLCV pipeline (Supplemental Fig. 2). Regardless of algorithm, models trained with demographic and vital variables showed a higher average test AUC as compared to models trained with solely the laboratory or advanced haematology variables (Table 2). LR scored the highest test AUC when trained on the demographic and vital variables alone (0.84, 0.79–0.89 95% CI). In comparison, the L1 and RF algorithms had similar test AUC scores when trained on all 70 variables, 0.85 (0.80–0.90 95% CI) and 0.84 (0.78–0.89 95% CI), respectively.

**Fig. 2:**
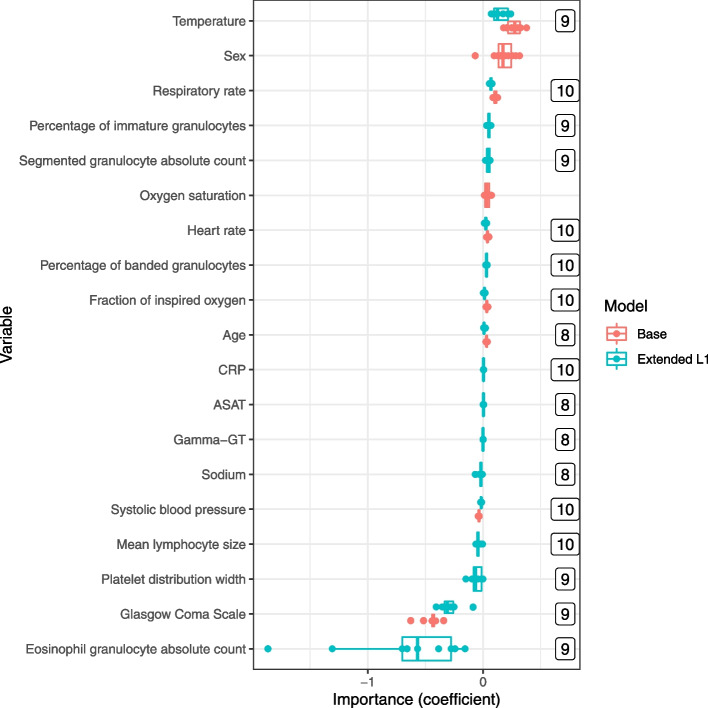
Variable importance of both the base (logistic regression with demographic and vital variables) and the extended L1 (L1 lasso with all data) models. Dots represent the learned coefficient in the trained models in the outer loop of the DLCV. The numbers indicate the number of times a variable was selected in the trained model in the outer loop of the DLCV. Only variables selected more than 7 times are shown

For each algorithm, the model with the highest test performance was used to investigate variable importance. Figure 1 shows ROC and PRC curves based on the aggregated 10 test folds predictions of the three selected models: base (logistic regression with demographic and vital data), extended L1 (lasso with all data) and extended RF (random forest trained with all data).

### Variable importance

**Fig. 3:**
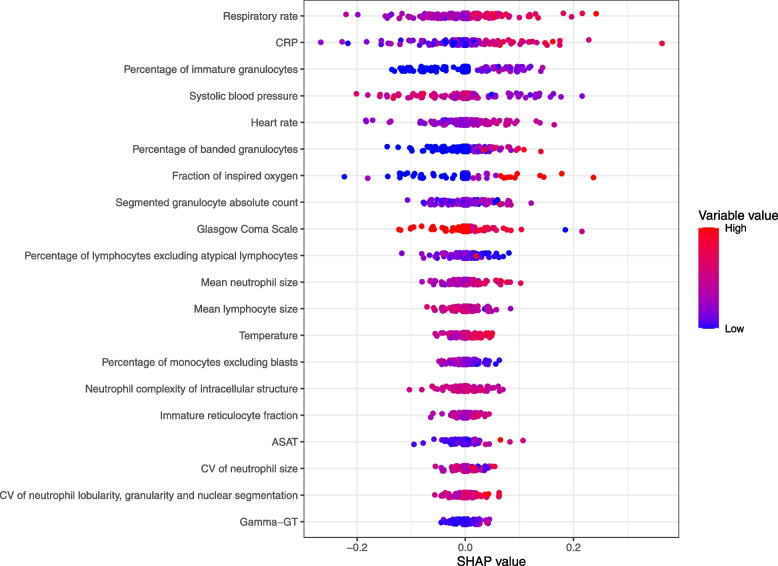
Aggregated SHAP values of the ten extended Random Forest models in the DLCV. The ten Random Forest models trained in the outer loop were used to predict probabilities of the outer fold samples. The first 20 variables sorted on absolute SHAP values are shown

Figure 2 shows the variable importance of both the base and the extended L1 models and Fig. 3 shows the SHAP values computed by the ten RF models. Of the nine demographic and vital variables, seven were also deemed as important by the extended L1 model, and 6 were also included in the 20 top variables of the extended RF model. Temperature, sex and GCS had on average the highest coefficient estimates for the base and extended L1 models, whereas the respiratory rate (rr), heart rate (hr) and systolic blood pressure (sbp) showed highest importance by the RF model. Of the laboratory variables, the L1 model selected glucose, ASAT, CRP, Gamma-GT and sodium in the DLCV, though with low coefficients. The extended RF identified Gamma-GT, ASAT and CRP. Concerning the advanced haematological variables, the L1 model identified percentage of immature granulocytes (pig), segmented granulocyte absolute count (seg), percentage of banded granulocytes (pbnd), mean lymphocyte size (lamn), platelet distribution width (pdw) and eosinophil granulocyte count (eos), as predictors for sepsis diagnosis, of which the last two had a negative coefficient and of which the latter showed the highest absolute coefficient of all variables. With SHAP, we found that the RF model also identified the same three variables as the L1 extended model; pig, pbnd and seg, with the same direction of importance: higher value is associated with sepsis.

## Discussion

As far as we know, this is the first study to investigate the use of an EAC as sepsis labels for the identification of new predictors with ML analyses using routine care data at the ED. For sepsis at the ED, there is no diagnostic gold standard and without such gold standard, developing a clinically valuable ML model is extremely challenging. In studies on ML and sepsis, the definition of sepsis is often based on suboptimal labels such as claims-based methods (e.g. ICD-10 coding) [14]. Alternatively, some ML models are based on clinical prediction scores such as qSOFA or SIRS, that were not designed for diagnostic purposes [27, 28]. Furthermore, these ill-suited methods may imply major limitations for the development of ML models at the ED, and consequently the identification of new predictors by ML. An EAC is likely the best option to define sepsis at the ED, since every member of the EAC is fully aware of the Sepsis-3 definition and will be able to apply this definition in clinical practice. Especially for relatively small datasets, such as our own, an EAC is executable and reliable.

Accurate sepsis diagnosis relies on accurate diagnostic tools to improve treatment strategies and outcome of septic patients. With machine learning, clinical diagnostic models can be developed by integrating big sets of routine care. Instead of ‘silver’ sepsis labels, we showed that in a relatively small dataset with high quality sepsis labels, ML can be used as method to identify new sepsis predictors on a wide variety of routine care data in the ED. Apart from the standard variables that are measured during an ED visit, we found multiple advanced haematology variables that show univariate diagnostic importance for sepsis diagnosis that is similar as compared to vital, demographic and standard laboratory variables. Moreover, known variables, such as granulocytes, and in particular the immature, banded granulocytes, were identified as important variables by both L1- and RF-extended models, serving as a positive control. Even though not many advanced haematology variables were selected by the machine learning algorithms, combining them with the routinely available variables resulted in a better performance, hinting at their diagnostic value.

The vital variables had a better diagnostic performance as compared to the laboratory and advanced haematological variables. In particular, hr and rr showed high univariate performance and were found to have high variable importance in both extended models. These results are in line with previous research and they are also used in multiple clinical prediction scores (e.g. (q)SOFA, MEWS) [5, 22, 29]. One reason that vital variables had an overall high performance may have been because the EAC was instructed to use the sepsis-3 criteria which includes sbp and rr. Therefore, the EAC may have quickly labelled patients as sepsis as these standard variables are available at the start of the clinical assessment.

Of the laboratory variables, only CRP showed high diagnostic performance in the univariate analysis. In the extended models, CRP was the second most important in the RF model, though CRP had a very low coefficient in the L1 model. CRP is a known maker for inflammation in blood and known to be associated with infection [30–32]. As the model evaluated many haematological variables associated with infection, the effect of CRP may have been reduced in the L1 model. Of the remaining laboratory variables, only ASAT or Gamma-GT showed marginally importance in the extended models, which are biomarkers for liver dysfunction. Although regarding the sepsis criteria, only bilirubin is used in the context of liver dysfunction [33].

The majority of the found advanced haematological variables by the extended models were related to granulocytes. Immature granulocytes are prematurely released from the bone marrow when the body’s immune response is active [34]. Research has shown that elevated values for ig and band neutrophils are associated with infection and can be an early sign of sepsis [35]. As result, these markers have been used for sepsis diagnosis as either a rule-in or rule-out test for early sepsis diagnosis [36]. Most interesting, the eosinophiles were negatively associated with sepsis in the L1 extended model. Wilar (2019) found the same association in neonatal sepsis as well as Abidi (2008) [37, 38]. As we removed correlated variables, the following variables were likely as predictive: immature granulocytes count (ig), banded granulocytes count (bnd) and percentage eosinophil granulocyte (peos).

As blood is drawn for every ED visit who visits our internal medicine department, complete laboratory data, including the advanced haematology variables, were available for almost all ED visits. Only a few visits missed 1 or 2 laboratory variables (data not shown). Although we imputed missing vital data points, all used vital variables had missing data < 1%, except for rr (28.6%), fio2 (15.6%), and spo2 (15.6%). Measuring variables related to the respiratory tract is most often neglected as it is time-consuming, especially in the ED where time is limited. A median rr value of 17 was used, which is representative for our internal medicine population. As rr was positively related to sepsis outcome, it is important that rr should accurately be measured during ED visit.

During model development, all data was used for training and testing in the DLCV scheme instead of a single train-test split. Also, no data was reused for hyperparameter optimization or testing in the DLCV scheme due to the inner fold and outer fold. Moreover, the variable importance was evaluated of each of the 10 trained DLCV models, instead of retraining a final model thereby reusing all data, hence introducing bias. Lastly, for each of the three selected models, we found a high AUC performance that strengthen the validity of the identified predictors. Even though a big proportion of the variables was found by both the L1 and RF models, both models also identified different variables. For example, the RF model could have identified non-linear relationships between variables, though interestingly these relationships were not supported by the SHAP values. However, evaluating variable importance does not imply causation, and is not a 1-to-1 comparison. Even though the same set of variables were identified as important by both L1 and RF algorithms, these predictors should first be evaluated as univariate predictors before being used in prediction models.

This study has several limitations. First, as this is a single centre study the results are not validated and may not be generalizable to other centres. Moreover, ED population are very diverse in terms of illness, age and secondary problems which may hamper validation of our results. Secondly, although we are convinced that an EAC is the optimal choice for labelling possible sepsis patients, an EAC is not perfect: patients will still be labelled based on the concept of sepsis of the expert. As a consequence, predictors known to be associated with sepsis, were also found by the algorithms. However, the advanced haematological variables were not available for the experts during the labelling process. Another limitation of an EAC is that it is labour intensive and may limit the reproducibility of our results. Nonetheless, there are semi-supervised ML models that would be able to label large datasets based on a couple of hundred EAC labelled patients [39]. We found that the final diagnosis sometimes remains uncertain for treating physicians at the ED. Therefore, it is possible that the final diagnosis as determined by the treating physician may differ from the EAC’s opinion. Future studies should be performed to explore these possibilities. Finally, we used ML to identify associations between variables and sepsis, but did not look into any causal relationships. Future studies should explore causal inference to evaluate the causal relationship to determine the dependence of the found variables with sepsis [40].

## Conclusions

Using ML and EAC generated labels we identified potential groups of predictors for sepsis diagnosis in routine EHR data, including new advanced haematological variables. Routinely collected variables from multiple sources already show predictive power for sepsis diagnosis, indicating that there is already value in data currently collected at the ED. Altogether, this method is an illustrative example of how machine learning can be utilized in data research. In our opinion, ML should not only be used to develop diagnostic tools, but also for other aspects of data research, such as the identification of diagnostic predictors and the related importance. This approach is not only applicable in the context of sepsis, but also for other syndromes that lack a reliable gold standard.

## Supplementary Information

**Additional file 1:** **Supplemental Table 1.** Definitions of all variables for the three variable types used in this study. **Supplemental Table 2.** Algorithms used in this study and the associated hyperparameters that were optimized in the double loop cross validation (DLCV). **Supplemental Table 3.** Immunocompromised definitions.** Supplemental Figure 1**. scheme of the double loop cross validation. HP: hyperparameter, CV: cross validation. **Supplemental Figure 2**. dendrogram of all 95 variables computed with Euclidean distance.

## Data Availability

The datasets generated and/or analyzed during the current study are not publicly available due to patient privacy but are available from the corresponding author (s.haitjema@umcutrecht.nl) upon reasonable request.
