# Supplementary material for: A machine learning approach using endpoint adjudication committee labels for the identification of sepsis predictors at the emergency department
Source: BMC Emerg Med. 2022 Dec 23;22:208. doi: 10.1186/s12873-022-00764-9 (PMC9784058; doi:10.1186/s12873-022-00764-9)

# Supplemental Materials

# Supplemental Table 1: definitions of all variables for the three variable types used in this study. Three groups of variables were defined: demographic and vital, laboratory and advanced haematological (sapphire). Area under the receiver operator curve (AUROC) indicate the univariate diagnostics performance computed on all data. CoV: Coefficient of Variance

| Type | Variable | Definition | AUROC |
| --- | --- | --- | --- |
| Demographic and vital | age | Age | 0.604 |
| Demographic and vital | sex | Sex | 0.512 |
| Demographic and vital | fio2 | Fraction of inspired oxygen | 0.589 |
| Demographic and vital | gcs | Glasgow Coma Scale | 0.607 |
| Demographic and vital | hr | Heart rate | 0.749 |
| Demographic and vital | rr | Respiratory rate | 0.748 |
| Demographic and vital | sbp | Systolic blood pressure | 0.675 |
| Demographic and vital | spo2 | Oxygen saturation | 0.562 |
| Demographic and vital | temp | Temperature | 0.658 |
| Laboratory | alat | ALAT | 0.551 |
| Laboratory | alk_phos | Alkaline phosphatase | 0.549 |
| Laboratory | asat | ASAT | 0.613 |
| Laboratory | creat | Plasma creatinine | 0.561 |
| Laboratory | crp | CRP | 0.749 |
| Laboratory | gamma_gt | Gamma-GT | 0.643 |
| Laboratory | glucose | Glucose | 0.590 |
| Laboratory | ld | Lactate dehydrogenase | 0.614 |
| Laboratory | potassium | Potassium | 0.523 |
| Laboratory | sodium | Sodium | 0.606 |
| Laboratory | urea | Urea | 0.646 |
| Sapphire | bas | Basophilic granulocyte absolute count | 0.559 |
| Sapphire | blst | Blast absolute count | 0.503 |
| Sapphire | bnd | Banded granulocyte absolute count | 0.698 |
| Sapphire | eos | Eosinophil granulocyte absolute count | 0.654 |
| Sapphire | hb | Hemoglobin | 0.584 |
| Sapphire | hdw | Hemobloin distribution width | 0.514 |
| Sapphire | ht | Hematocrit | 0.578 |
| Sapphire | ig | Immature granulocyte absolute count | 0.688 |
| Sapphire | irf | Immature reticulocyte fraction | 0.647 |
| Sapphire | lacv | CV of variance of lymphocyte size | 0.59 |
| Sapphire | lamn | Mean lymphocyte size | 0.655 |
| Sapphire | licv | CV of lymphocyte complexity of intracellular structure | 0.532 |
| Sapphire | limn | Lymphocyte complexity of intracellular structure | 0.598 |
| Sapphire | lym | Lymphocyte absolute count | 0.567 |
| Sapphire | lyme | Lymphocyte (excluding atypical lymphocytes) absolute count | 0.567 |
| Sapphire | mch | Mean corpuscular hemoglobin | 0.533 |
| Sapphire | mchc | Mean corpuscular hemoglobin concentration | 0.556 |
| Sapphire | mchcr | Mean corpuscular HGB concentration per reticulocyte | 0.597 |
| Sapphire | mchr | Mean corpuscular HGB per reticulocyte | 0.58 |
| Sapphire | mcv | Mean corpuscular volume | 0.513 |
| Sapphire | mcvr | Mean corpuscular volume of reticulocytes | 0.546 |
| Sapphire | mon | Monocyte absolute count | 0.456 |
| Sapphire | mone | Monocyte (excluding blasts) absolute count | 0.547 |
| Sapphire | mpv | Mean platalet volume | 0.56 |
| Sapphire | nacv | CV of neutrophil size | 0.575 |
| Sapphire | namn | Mean neutrophil size | 0.685 |
| Sapphire | ndcv | CV of neutrophil depolarization | 0.552 |
| Sapphire | ndmn | Neutrophil lobularity/granularity and nuclear lobularity | 0.571 |
| Sapphire | neu | Neutrophil granulocytes absolute count | 0.678 |
| Sapphire | nfcv | CV of fluorescence for DNA/RNA staining in neutrophils | 0.52 |
| Sapphire | nfmn | Fluorescence for DNA/RNA staining in neutrophils | 0.59 |
| Sapphire | nicv | CV of neutrophil complexity of intracellular structure | 0.618 |
| Sapphire | nimn | Neutrophil complexity of intracellular structure | 0.573 |
| Sapphire | npcv | CV of neutrophil lobularity, granularity and nuclear segmentation | 0.615 |
| Sapphire | npmn | Neutrophil lobularity, granularity and nuclear segmentation | 0.66 |
| Sapphire | nrbc | Nucleated Red Blood Cells (NRBC) absolute count | 0.505 |
| Sapphire | pbas | Percentage of basophilic granulocytes | 0.619 |
| Sapphire | pblst | Percentage of blasts | 0.501 |
| Sapphire | pbnd | Percentage of banded granulocytes | 0.702 |
| Sapphire | pct | Plateletcrit | 0.543 |
| Sapphire | pdw | Platelet distribution width | 0.553 |
| Sapphire | peos | Percentage of eosinophilic granulocytes | 0.726 |
| Sapphire | phpo | Percent of RBCs with HGB concentration less than 28 g/dL | 0.525 |
| Sapphire | phpr | Percent of RBCs with HGB concentration more than 41 g/dL | 0.502 |
| Sapphire | picv | CV of platelet complexity of intracellular structure | 0.588 |
| Sapphire | pig | Percentage of immature granulocytes | 0.684 |
| Sapphire | pimn | Platelet complexity of intracellular structure | 0.539 |
| Sapphire | plt | Platelet absolute count | 0.524 |
| Sapphire | plti | Platelet count by impedance | 0.529 |
| Sapphire | plto | Platelet count by optics | 0.524 |
| Sapphire | plym | Percentage of lymphocytes | 0.658 |
| Sapphire | plyme | Percentage of lymphocytes excluding atypical lymphocytes | 0.659 |
| Sapphire | pmac | Percentage of red blood cells with volume greater than 120 fL | 0.513 |
| Sapphire | pmic | Percentage of red blood cells with volume less than 60 fL | 0.578 |
| Sapphire | pmon | Percentage of monocytes | 0.669 |
| Sapphire | pmone | Percentage of monocytes excluding blasts | 0.67 |
| Sapphire | pneu | Percentage neutrophilic granulocytes | 0.704 |
| Sapphire | pnrbc | Percentage of nucleated red blood cells per 100 white blood cells | 0.505 |
| Sapphire | ppcv | CV of platelet lobularity/granularity | 0.576 |
| Sapphire | ppmn | Platelet lobularity/granularity | 0.563 |
| Sapphire | pretc | Percentage of reticulocytes | 0.552 |
| Sapphire | prp | Percentage of reticulated platelets | 0.644 |
| Sapphire | pseg | Percentage of segmented granulocytes | 0.528 |
| Sapphire | pvlym | Percentage of atypical lymphocytes | 0.487 |
| Sapphire | rbcfcv | CV of FL1 signal (presence of NRBCs) | 0.518 |
| Sapphire | rbcfmn | Mean of FL1 signal (presence of NRBCs) | 0.518 |
| Sapphire | rbci | Red blood cell count by impedance | 0.568 |
| Sapphire | rbcicv | CV of red blood cell complexity of intracellular structure | 0.537 |
| Sapphire | rbcimn | Red blood cell complexity of intracellular structure | 0.542 |
| Sapphire | rbco | Red blood cell count by optics | 0.566 |
| Sapphire | rdw | Red blood cell distribution width | 0.583 |
| Sapphire | retc | Reticulocyte absolute count | 0.535 |
| Sapphire | seg | Segmented granulocyte absolute count | 0.613 |
| Sapphire | vlym | Atypical lymphocyte absolute count | 0.487 |
| Sapphire | wbc | White blood cell absolute count | 0.652 |
| Sapphire | wvf | White blood cell viability fraction | 0.512 |

# Supplemental Table 2: algorithms used in this study and the associated hyperparameters that were optimized in the double loop cross validation (DLCV). Configuration of hyperparameters may improve the performance of machine learning algorithms. Configurations are shown of both the Lasso (L1) and Random Forest (RF) machine learning algorithms that were optimized in he DLCV

| Algorithm | Category | Hyperparameter |
| --- | --- | --- |
| Logistic regression (LR) | Conventional | - |
| Lasso (L1) | Regularization | Lambda: from 0 to 1 |
| Random Forest (RF) | Non-linear trees | ntree: {25, 50, 75}  mtry: {2, 4, 6}  min_n: {50, 75, 100} |

**Supplemental Table 3: immunocompromised definitions.** A patient was considered immunocompromised if the patients met one of the conditions described below.

| Neutropenia | Neutrophil cell count <0.5 x 10^9^ during presentation at the emergency department. |
| --- | --- |
| Autologous stem-cell Tx within last 6 months | Autologous stem-cell transplantation within 6 months previous to the emergency department visit. |
| Allogenic stem-cell Tx within last 6 months | Allogenic stem-cell transplantation within 6 months previous to the emergency department visit. |
| Solid organ Tx (using immunosuppressives within last 6 months) | Patient with a history of solid organ transplantation (e.g. heart, lung, liver, kidney etc.) and using immunosuppressives within last 6 months. |
| Corticosteroids equivalent of prednisolone dose > 700mg total and/or 20 mg per day | Usage of a cumulative dose of prednisone >700 mg and/or a dose of 20mg prednisone per day (or equivalent corticosteroid dosage) previous to emergency department visit. |
| Anti-CD-20 therapy within last six months. | Treatment within last six months with one of the following medicaments: rituximab, ocrelizumab, veltuzumab, obinutuzumab or ofatumumab. |
| Biologicals (anti-TNF, anti-IL-1, anti-IL-6, nataluzimab etc) within the last 6 months | Treatment within last six months with a biological, including but not limited to: anti-TNF, anti-IL-1, anti-IL-6 |
| MTx/azathioprine/6MP within last 6 months | Treatment within the last six months with one of the following medicaments: methotrexate, purine antagonists |
| (Functional) hypo/asplenia | Asplenia at the time of visiting the emergency department. This condition can be primary or acquired, for example due to surgery or multiple splenic infarctions |
| Primary immunodeficiency | A primary immunodeficiency diagnosed by a paediatrician, internist or immunologist, documented in the patient’s medical history. |
| CD4-penia <200/mm3 | During the emergency department visit a documented CD4-count <200/mm^3^ |
| Hypogammaglobulinemia | During the emergency department visit or in the patient’s medical history a documented shortage of gamma globulins    *Please note: IgA-deficiency is covered by this* |

**Supplemental Figure 1: scheme of the double loop cross validation. HP: hyperparameter, CV: cross validation.** *: data was stratified on both label and patient ID.


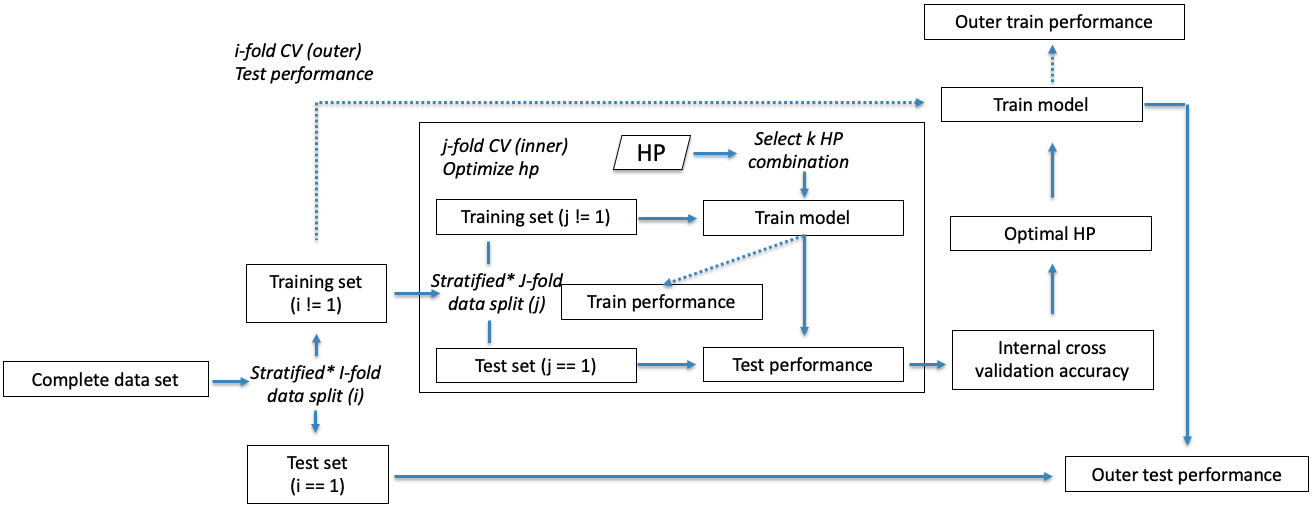


**Supplemental Figure 2: dendrogram of all 95 variables computed with Euclidean distance.** Length of each node depicts the similarity between variables. The blue circle represents the 0.80 cut-off. Colours represent the variable groups: vital (purple), demographic (red), laboratory (green) and advanced haematological (blue).


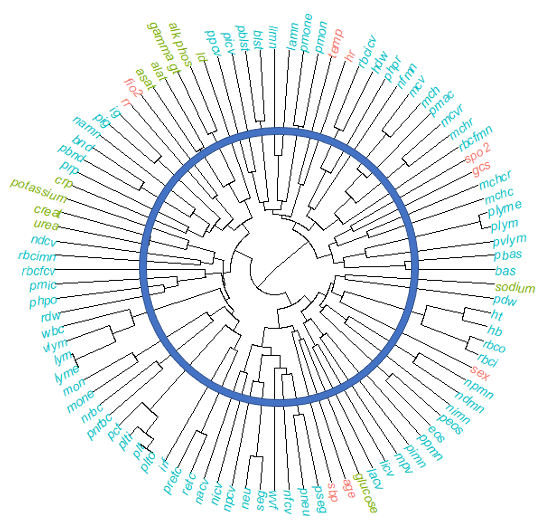

Supplement: Supplementary file 1 — Additional file 1: Supplemental Table 1. Definitions of all variables for the three variable types used in this study. Supplemental Table 2. Algorithms used in this study and the associated hyperparameters that were optimized in the double loop cross validation (DLCV). Supplemental Table 3. Immunocompromised definitions. Supplemental Figure 1. scheme of the double loop cross validation. HP: hyperparameter, CV: cross validation. Supplemental Figure 2. dendrogram of all 95 variables computed with Euclidean distance. [file 12873_2022_764_MOESM1_ESM.docx]
